# The Effect of Microwave Baking Conditions on the Quality of Biscuits and the Control of Thermal Processing Hazards in the Maillard Reaction

**DOI:** 10.3389/fnut.2022.825365

**Published:** 2022-02-25

**Authors:** Lu Dong, Caiyi Qiu, Fan Wei, Zhenting Yu, Yan Zhang, Shuo Wang

**Affiliations:** Tianjin Key Laboratory of Food Science and Health, School of Medicine, Nankai University, Tianjin, China

**Keywords:** microwave-baking, thermal processing hazards, sensory characteristics, microwave time, microwave power

## Abstract

To reduce thermal processing hazards (TPHs), microwave baking has been extensively used in food thermal processing. In this study, the influence of microwave power and microwave time on the formation of TPHs and their precursors was explored in microwave-baked biscuits. The results indicated that the content of acrylamide, 5-hydroxymethylfurfural, methylglyoxal, and 3-deoxyglucosone increased linearly with the extension of microwave time (2, 2.5, and 3 min) and microwave power (440, 480, and 520 W). There was a significant correlation between the four TPHs. 3-Deoxyglucosone may directly or indirectly participate in the formation of the other three TPHs. The relationship between TPH levels with some heat-induced sensory characteristics was analyzed. The correlation between the sensory characteristics and the content of TPHs is L^*^ > a^*^ > hardness > Water activity (AW). The correlation coefficients between L^*^ value and the four TPHs are −0.950, −0.891, −0.803, and −0.985. Furthermore, the content of TPHs produced by traditional baking and microwave baking under the same texture level was compared. Compared with traditional baking (190°C, 7 min), microwave baking at 440 W for 3 min successfully decrease methylglyoxal, 3-Deoxyglucosone, acrylamide, and 5-hydroxymethylfurfural content by 60.75, 30.19, 30.87, and 61.28%, respectively. Traditionally baked biscuits, which had a more obvious color, as characterized by lower L^*^ value, larger a^*^ and b^*^ values, are more susceptible to the formation of TPHs. Therefore, microwave baking can reduce the generation of TPHs.

## Introduction

Biscuits are favored as a snack food as they are rich in nutrients, easy to store and carry, and long lasting (1). The main ingredients are wheat flour, sugar, and fat, which provide a material basis for the Maillard reaction. The traditional processing method used for biscuits is baking in hot air (2). During the baking process, sugar, protein, and fat interact with each other to give the unique sensory characteristics of biscuits, including flavor, appearance, color, and texture (3, 4). The traditional baking will also lead to the inevitable damage to the nutrients in food. Meanwhile, a variety of thermal processing hazards (TPHs) are generated, such as acrylamide (AM), 5-hydroxymethylfurfural (HMF), methylglyoxal (MG), and 3-deoxyglucosone (3-DG), which have the potential to endangers food safety (5–8). Therefore, in the recent years, the reduction and control of food TPHs have become a hot topic in the field of food safety research. As more and more new thermal processing technologies are applied to food processing, new ideas for the reduction and control of food TPHs are generated.

Microwave methods are widely used in the food industry and in domestic cooking, including for drying, sterilization, baking, and heating (9, 10). The principle of microwave generates heat by the vibration and collision of polar molecules throughout the entire food. Microwave heating results in a faster heating rate and more uniform heating (11). The temperature of the outer surface of the food is lower than that of traditional baking, which is not conducive to the occurrence of Maillard reaction and can provide control over the formation of TPHs. At present, the research of microwave baking is mainly focused on the effect of microwave heating on dough texture and color, the loss of water during heating, and the change of functional characteristics of dough components (12–14), However, there are few studies on the influence of microwave on the formation of TPHs in biscuits. AL-Ansi et al. (15) reported that fennel or nigella to biscuits under microwave baking conditions reduced AM production more than traditional baking. Some report's results indicate that microwaves can also promote Maillard reactions, similar to traditional heating. Ye et al. found that the content of AM and MG in potato chips baked in a microwave at 700 W for 3 min was higher than that in chips fried at 160°C for 7 min and 180°C for 6 min (16). Juodeikiene et al. reported that the content of AM in corn products that were subjected to vacuum microwave treatment was up to 74.3% higher than in corn products that were subjected to infrared irradiation (17). This raises questions of whether the heat energy given to the food by the two processing methods is the same. However, this is rarely addressed in research studies. It was speculated that microwaves result in a rapid temperature increase, which results in the rapid attainment of high temperature and the generation of TPHs (16). However, it is wrong to directly compare the effects of microwave and traditional heating methods on TPHs. Instead, the same heat energy generated by the processing methods should be the basis for comparing the effects of TPHs formation.

The changes in the sensory characteristic of food ingredients are closely related to the formation of TPHs. One potential solution is the evaluation of the influence of different processing methods on the formation of TPHs based on the same specific sensory characteristics of the food matrix. In the study of the influence of microwave on the sensory characteristics of French fries and the formation of AM, it was found that compared with fried French fries (180°C for 8 min), the content of AM in microwave fries was 37–83% lower with the microwave power of 315, 430, and 600 W, along with higher moisture content and better fracturability (18). Gökmen et al. (19) established an AM prediction model based on the changes in the color of the biscuit surface.

The formation of TPHs is also closely related to the content of TPHs precursors in the food. It is found that the production of MG (*r* = 0.987) and G (*r* = 0.997) is linearly related to the content of AM in traditionally baked biscuits, but was not correlated with the formation of HMF (20). The amount of HMF generated in the fried fries as a model food can be used to predict the amount of AM generated, with a correlation coefficient of *r* = 0.922 (16). At present, there is a serious lack of comprehensive reports on the influence of microwave heating on the formation mechanism and sensory characteristics of TPHs in food.

In this study, the influence of microwave power (440, 480, and 520 W) and microwave time (2, 2.5, and 3 min) applied on the formation of TPHs and their precursors were explored, along with the correlation between the sensory characteristics and TPHs of biscuits. Furthermore, the content of TPHs produced by traditional baking and microwave baking under the same texture level was compared. The impact of microwave baking on biscuits quality from the perspectives of food safety and sensory properties was explored, with the aim of providing a theoretical basis for the application of microwave baking technology in food processing.

## Materials and Methods

### Materials and Reagents

Standards of MG, HMF, AM, and *o*-phenylenediamine were supplied by Sigma-Aldrich (Mosby, USA). The standard for 3-DG was obtained from TRC (Toronto, Canada). Acetonitrile (HPLC grade) was purchased from Merck (Darmstadt, Germany). Water was prepared using a Milli-Q integral purification system (Molsheim, France). Oasis HLB solid-phase extraction (SPE) cartridges (6 mL, 200 mg) were obtained from Waters (Milford, USA). All other reagents were of analytical reagent grade. The ingredients for making biscuits were purchased from a local market.

### Biscuit Preparation

The dough was composed of 80 g of sucrose, 40 g of shortening, 25 g of butter, 1.1 g of sodium bicarbonate, 0.9 g of NaCl, 13 g of whole milk powder, approximately 40 g of egg, and 300 g of flour. First, the shortening and butter were creamed and mixed with sucrose and egg. Then, sodium bicarbonate and NaCl were added and mixed. Finally, the whole milk powder, flour, and approximately 30 ml of water were added and mixed evenly. The dough was rolled out to a disk with a diameter and thickness of 4 and 2 mm, respectively. The microwave power (440, 480, and 520 W) and microwave time (2, 2.5, and 3 min) were chosen based on the findings of a preliminary experiment, where biscuits baked in a microwave at a power of 520 W for 3 min produced excessively burnt samples. Traditional baking at 190°C for 7 min was used as the control.

### Biscuit Pre-treatment

In total, 1 g of ground biscuit sample was dispersed in 9 ml of Milli-Q water, and 500 μl of 1 mg/L ^13^C_3_-AM internal standard working solution was added. The solution was centrifuged at 6,000 × g for 30 min at 4°C and the supernatant was obtained. Then, 20 ml *n*-hexane and 0.5 mL each of Carrez reagent and Carrez division reagent were added to degrease and remove proteins. Subsequently, the solution was centrifuged at 6,000 × g for 30 min at 4°C to obtain the supernatant, which was used for the subsequent treatment and analysis of different TPHs.

### Quantitation of αDCs by HPLC-MS/MS

*o*-Phenylenediamine (40 mg/ml) was used to derivatize α-dicarbonyl compounds (αDCs) at 60°C for 2 h in 1 ml of the supernatant obtained from pretreatment. After derivatization, concentration and purification were performed by Oasis HLB SPE cartridges (Waters, New Castle, Pennsylvania, USA) followed by filtration through a 0.22 μm nylon filter for Liquid chromatography tandem mass spectrometry (LC-MS/MS) analysis. Then, the content of αDCs in the filtrate was analyzed using an Agilent 1200 LC system coupled to an Agilent 6410 triple-quadrupole MS system (Agilent, USA) equipped with a Polar-RP 80Å column (2 × 150 mm, 4 μm). Methanol (A) and 0.1% formic acid (B) were used as the mobile phase, with a stepwise gradient procedure (0 min, 20%A; 0–15 min, 30% A; 15–30 min, 80% A; and 30–35 min, 15% A) at a flow rate of 0.3 L/min. Positive ion mode was used to operate Electrospray ionization mass spectrometry (ESI-MS) and αDCs were identified by selected ion monitoring. Nitrogen was used as the nebulizer, drying, and collision gases. The nebulizer pressure was 40 psi, the capillary voltage was 4 kV, and the drying gas flow rate was 600 L/h at 350°C. The product ions of quinoxaline derivatives at m/z 235, 199 (3-DG), and 145, 118 (MG) were used for quantification and confirmation, respectively. The limit of detection (LOD) of 3-DG and MG in the biscuits was 0.503 and 0.089 μg/kg, respectively, and the limit of quantification (LOQ) was 1.675 and 0.297 μg/kg, respectively. Recoveries of 3-DG and MG were 84.43–103.78% and 85.25–101.01%, respectively, and the relative SD (RSD) was 2.45–4.37% and 6.76–9.33%, respectively.

### Quantification of AM by UPLC-MS/MS

The supernatant obtained from pre-treatment methods was purified through an Oasis MCX 3 ml (60 mg) column (Waters, New Castle, Pennsylvania, USA) with activation and equilibration performed with 5 ml of methanol and 5 ml of Mill-Q water before using. The target was then eluted with 4 ml of methanol, and the eluent was collected. The samples were filtered through a 0.22 μm nylon filter and subjected to UPLC-MS/MS analysis. Water Xevo TQ-S Micro UPLC-MS/MS (New Castle, Pennsylvania, USA) was used to analyze the content of AM. A Symmetry C18 column (5 μm, 4.6 × 150 mm) was used with a column oven at 30°C to separate the sample. Methanol (A) and 0.1% formic acid (B) were used as the mobile phase with a stepwise gradient procedure (0–15 min, 5% A; 9–11 min, 5% A; and 11.5–13 min, 5% A) at a flow rate of 0.3 ml/min. Positive ion mode was set to operate the ESI-MS and AM was identified by multireaction monitoring. Nitrogen was used as a nebulizer, drying, and collision gases. The nebulizer gas flow rate was 60 L/h, the capillary voltage was 3.5 kV, and the drying gas flow rate was 800 L/h at 500°C. The product ions of 55 and 48 m/z were used for quantification and confirmation, and a seven-point linear calibration curve was established in the range of 1–100 ng/ml (*R*^2^ > 0.9999). The LOD was 0.519 μg/kg, and the LOQ was 1.633 μg/kg. The recoveries were stable in the range of 83.74–93.60%, and the RSD was 0.53–8.21%, which proved that the method was accurate and accurate and suitable for the determination of AM content in microwave-baked biscuits.

### Quantification of HMF by HPLC

The pretreatment method for HMF was the same as for AM. HMF was determined using a Shimadzu HPLC system LC-20A (Kyoto, Japan) with a UV detector in accordance with the previous method, but with some modifications (21). A HYPERSIL ODS-2 C18 column (4.6 × 250 mm, 5 μm) was used to separate the sample with the column oven at 30°C. The mobile phase was acetonitrile: water (5 :95, v/v) at a flow rate of 0.6 ml/min. The injection volume was 20 μl and the UV detection wavelength was 284 nm. The LOD and LOQ in the biscuits were 0.01 mg/kg and 0.04 mg/kg, respectively. The recoveries and RSD were 82.53–88.94% and 2.76–6.19%, respectively.

### Detection of Water Activity (AW)

The AW of the microwave-baked biscuits was analyzed directly with an AW indicator (Novasina LabAwift-aw, Lachen, Switzerland).

### Color Analysis

A Konica Minolta Spectrophotometer CM-5 colorimeter (Tokyo, Japan) was used to perform the color measurements of biscuits. L^*^ (lightness), a^*^ (greenness/redness), and b^*^ (blueness/yellowness) were used to express the color of biscuits. Each sample was measured six times and the average of the readings was calculated.

### Texture Analysis

The texture of biscuits was measured with a TA-XT plus texture analyzer (Stable Micro Systems, UK) coupled with an HDP/3PB probe. The following settings were used: pre-test speed, 3 mm/s; test speed, 3 mm/s; posttest speed, 10 mm/s; press distance, 8 mm; trigger force, 5 g. Each sample was measured six times and the average reading of the texture indices (hardness and fracturability) was calculated for one sample.

### Sensory Evaluation and Fuzzy Reasoning

The sensory evaluation method was referred to the previous study with some modification (19). Sensory attributes (appearance, color, taste, and texture) of traditionally baked biscuits and microwave baked biscuits were evaluated with four different expressions (excellent, good, fair, and poor) by 10 postgraduates with food sensory evaluation experience as panelists who were participants in this study. The fuzzy reasoning method was used to evaluate the overall acceptability. Fuzzy set sensory attributes set (PA) was used for sensory attributes, including elements of appearance, color, taste, and texture. The membership degree was 0.2, 0.3, 0.2, and 0.3, respectively. Another fuzzy set, preference degree (PB), was established to represent the preference degree, and consisted of excellent, good, fair, and poor elements, with scores of 4, 3, 2, and 1, respectively.

### Statistical Analysis

All the analyses were performed in triplicate, with the data were recorded as the mean ± standard deviation. GraphPad Prism 8.0.2 (GraphPad Software, San Diego, California, USA) was used to perform one-way ANOVA and Tukey's test; statistical significance was determined for *p*-values of ≤ 0.05. The correlation between TPH level and sensory characteristics was evaluated by the Pearson's coefficient.

## Results

### Thermal Processing Hazards Content in Microwave Baked Biscuits

**Figure 1:**
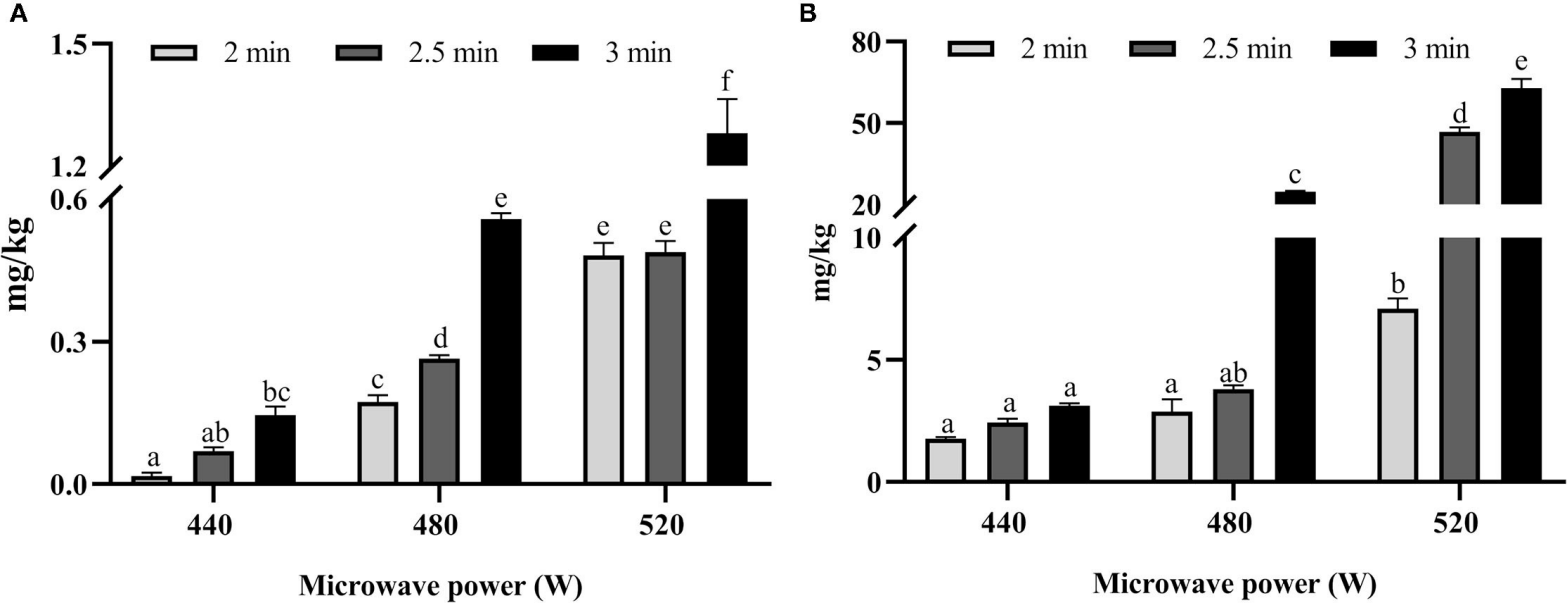
The content of methylglyoxal (MG) **(A)** and 3-deoxyglucosone (DG) **(B)** in microwave-baked biscuits prepared under different conditions. Different lowercase letters indicate significant differences (at *p* < 0.05) between different cooking conditions.

The content of MG and 3-DG in microwave-baked biscuits is shown in Figures 1A,B and ranges from 0.019 to 1.262 mg/kg and from 1.781 to 62.956 mg/kg, respectively. With the exception of the 3-DG content in biscuits baked in the microwave at 440 W for all times, the MG and 3-DG content were significantly (*p* < 0.05) increased as the microwave time was prolonged and the power was increased.

**Figure 2:**
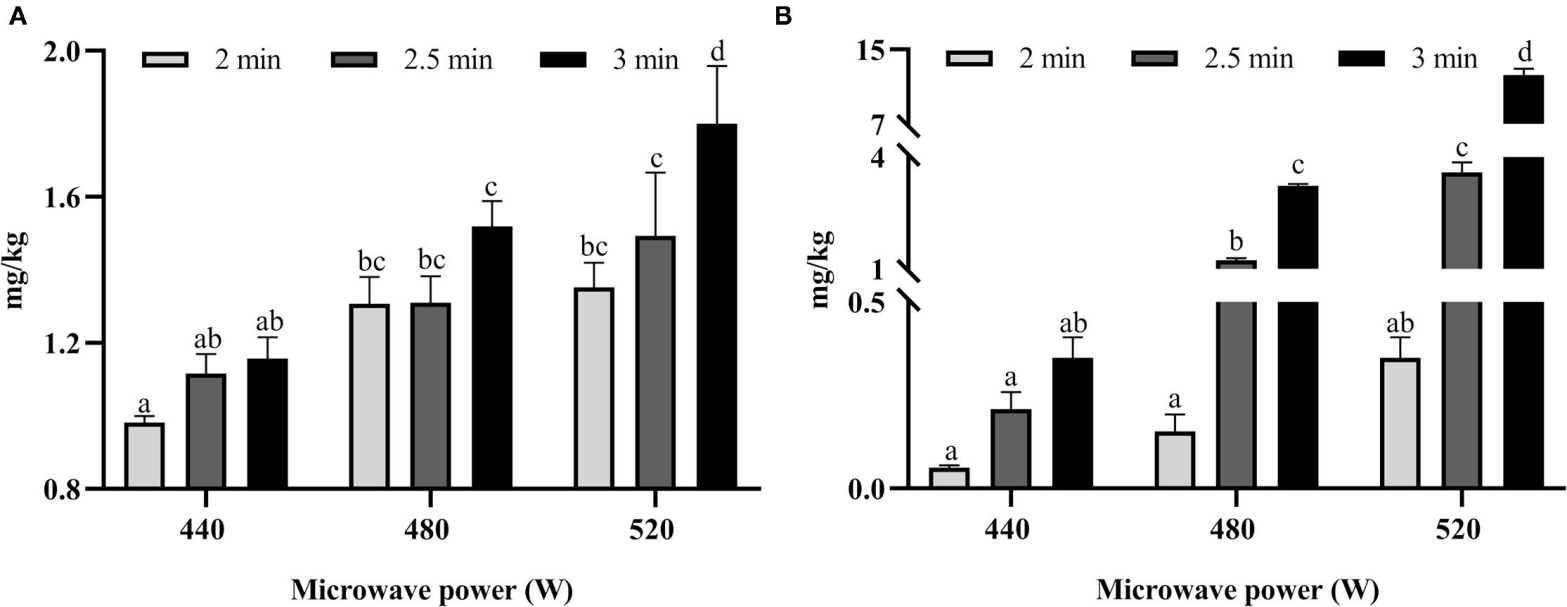
The content of acrylamide (AM) **(A)** and hydroxymethylfurfural (HMF) **(B)** in microwave-baked biscuits prepared under different conditions. Different lowercase letters indicate significant differences (at *p* < 0.05) between different cooking conditions.

As shown in Figures 2A,B, the concentrations of AM and HMF were ranged from 0.975 to 1.801 mg/kg and from 0.060 to 11.658 mg/kg, respectively. With the exception of the AM and HMF content in biscuits baked in the microwave at 440 W for all times, the contents were also significantly (*p* < 0.05) increased as the microwave time was prolonged and power was increased.

### Sensory Characteristics in Microwave Baked Biscuits

As shown in Table 1, the sensory characteristics of AW, texture, and color were measured. The AW and a^*^ values were decreased as the time and power were increased. The hardness and L^*^ values were increased as the time and power increased. The fracturability of biscuits was increased as the microwave time increased for microwave power of 440 and 480 W, and the fracturability of biscuits was decreased as the microwave time increased at the power of 520 W. The changes in b^*^ values followed an opposite trend to the trend of the change in fracturability.

**Table 1:** AW, texture, and color characteristics of microwave baked biscuits.

**Samples**	**AW**	**Hardness/g**	**Fracturability/mm**	L*	a*	b*
440 W 2 min	0.71 ± 0.01^a^	1916.97 ± 297.46^a^	4.50 ± 0.30^a^	81.93 ± 0.11^a^	1.61 ± 0.10^a^	27.90 ± 0.36^b^
440 W 2.5 min	0.55 ± 0.01^b^	2621.04 ± 87.54^ab^	5.01 ± 0.19^ab^	81.44 ± 0.11^ab^	2.30 ± 0.07^b^	27.84 ± 0.34^b^
440 W 3 min	0.44 ± 0.01^c^	3705.63 ± 375.02^cd^	5.44 ± 0.35^bd^	81.66 ± 0.05^a^	2.22 ± 0.05^b^	27.50 ± 0.01^bc^
480 W 2 min	0.45 ± 0.02^c^	3177.38 ± 204.46^bc^	5.24 ± 0.26^ac^	80.71 ± 0.15^ac^	2.16 ± 0.05^b^	27.07 ± 0.04^bc^
480 W 2.5 min	0.32 ± 0.02^d^	4258.49 ± 371.29^de^	5.55 ± 0.32^bd^	80.56 ± 0.09^ac^	2.41 ± 0.05^b^	26.97 ± 0.06^c^
480 W 3 min	0.10 ± 0.01^f^	5197.56 ± 252.35^ef^	6.04 ± 0.26^cd^	79.13 ± 0.19^c^	2.99 ± 0.03^c^	26.85 ± 0.21^c^
520 W 2 min	0.20 ± 0.01^e^	4618.96 ± 222.32^de^	6.26 ± 0.03^d^	80.00 ± 0.12^bc^	2.44 ± 0.04^b^	26.95 ± 0.05^c^
520 W 2.5 min	0.10 ± 0.01^f^	5235.44 ± 29.69^ef^	5.88 ± 0.09^bd^	77.23 ± 0.31^d^	3.77 ± 0.16^d^	26.70 ± 0.16^c^
520 W 3 min	0.09 ± 0.01^f^	5950.45 ± 477.01^f^	5.16 ± 0.28^ac^	65.99 ± 1.33^e^	9.28 ± 0.10^e^	29.39 ± 0.45^a^

*Different lowercase letters indicate significant differences (at p < 0.05) between different cooking condition*.

*Superscript lowercase of a–f indicate the sensory characteristics with significant differences (at p < 0.05) between different cooking conditions*.

### Correlation Analysis and Pearson Analysis of the TPHs Content With Sensory Characteristic

**Figure 3:**
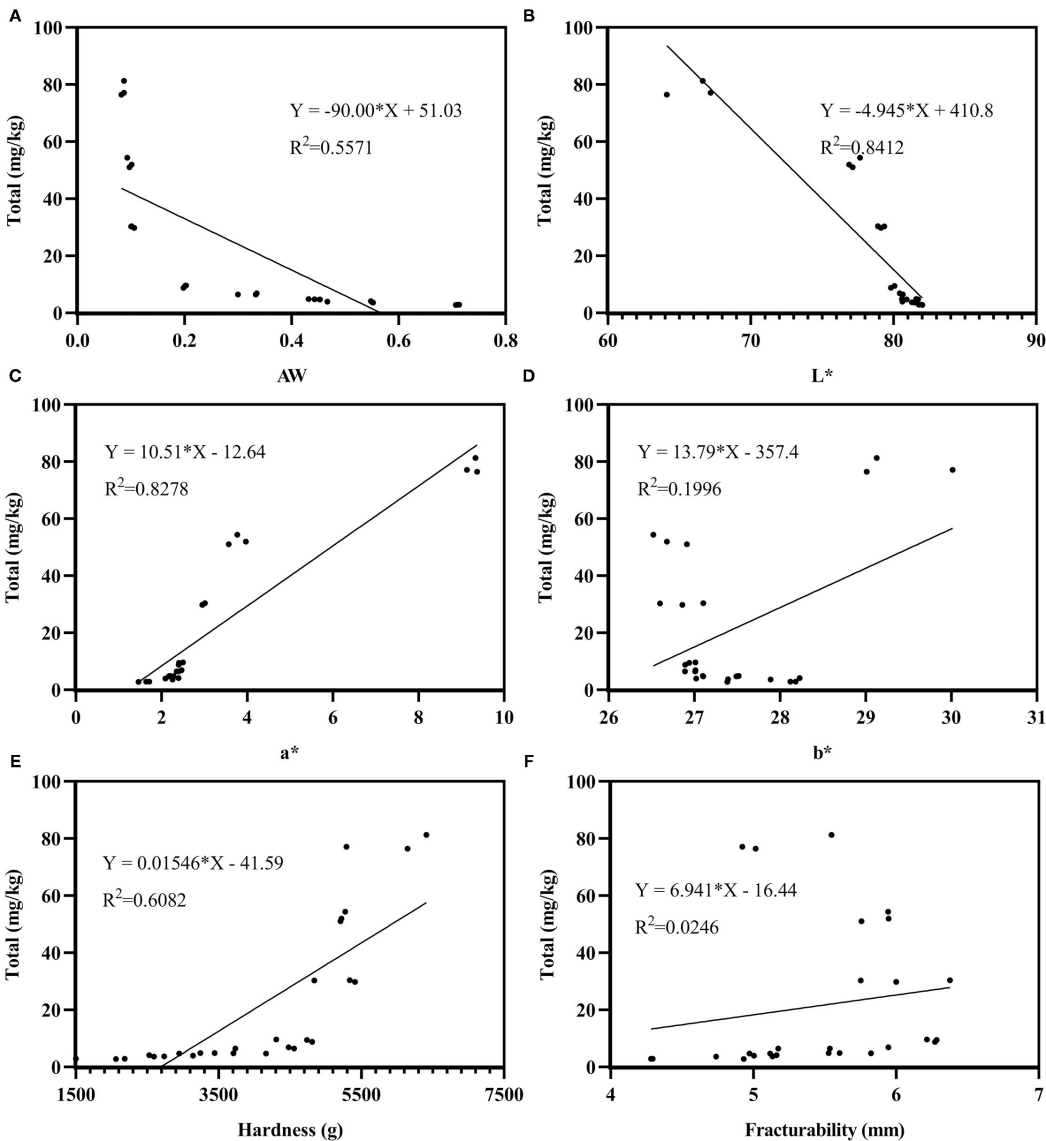
Linear regression lines between total thermal processing hazards (TPHs) and AW **(A)**, L* **(B)**, a* **(C)**, b* **(D)**, hardness **(E)**, and fracturability **(F)** in microwave-baked biscuits.

The TPH contents and sensory characteristics were determined by Pearson analysis. The Pearson coefficients between TPHs and sensory characteristics are in given in Table 2. The TPH contents were negatively (*p* < 0.01) correlated with AW and L^*^ value, but positively (*p* < 0.01) correlated with a^*^ value and hardness. The correlation between sensory characteristics and the formation of TPHs was L^*^ > a^*^ > hardness > AW > b^*^ > fracturability. However, both the b^*^ value and fracturability have a poor correlation with TPHs formation. Moreover, the linear regression lines between total THPs and sensory characteristics are shown in Figure 3. L^*^ value is also the highest correlation with total content of TPHs among of sensory characteristics (*r* = −0.917, *p* < 0.01).

**Table 2:** The correlation coefficients between thermal processing hazards (THPs) and sensory characteristics of microwave-baked biscuits.

	**MG**	**DG**	**AM**	**HMF**	**AW**	**L**	**a**	**b**	**Hardness**	**Fracturability**	**Total THPs**
MG	1	0.881^**^	0.879^**^	0.944^**^	−0.796^**^	0.950^**^	0.936^**^	0.485^*^	0.839^**^	0.255	0.905^**^
DG	0.881^**^	1	0.822^**^	0.910^**^	−0.752^**^	−0.891^**^	0.882^**^	0.405^*^	0.778^**^	0.177	0.998^**^
AM	0.879^**^	0.822^**^	1	0.809^**^	−0.859^**^	−0.803^**^	0.790^**^	0.285	0.836^**^	0.388^*^	0.834^**^
HMF	0.944^**^	0.910^**^	0.809^**^	1	−0.632^**^	−0.985^**^	0.986^**^	0.651^**^	0.707^**^	0.000	0.936^**^
AW	−0.796^**^	−0.752^**^	−0.859^**^	−0.632^**^	1	0.619^**^	−0.595^**^	0.077	−0.957^**^	−0.687^**^	−0.746^**^
L	−0.950^**^	−0.891^**^	−0.803^**^	−0.985^**^	0.619^**^	1	−0.992^**^	−0.653^**^	−0.696^**^	−0.008	−0.917^**^
a	0.936^**^	0.882^**^	0.790^**^	0.986^**^	−0.595^**^	−0.992^**^	1	0.699^**^	0.677^**^	−0.014	0.910^**^
b	0.485^*^	0.405^*^	0.285	0.651^**^	0.077	−0.653^**^	0.699^**^	1	0.029	−0.541^**^	0.447^*^
Hardness	0.839^**^	0.778^**^	0.836^**^	0.707^**^	−0.957^**^	−0.696^**^	0.677^**^	0.029	1	0.614^**^	0.780^**^
Fracturability	0.255	0.177	0.388^*^	0.000	−0.687^**^	−0.008	−0.014	−0.541^**^	0.614^**^	1	0.157
Total THPs	0.905^**^	0.998^**^	0.834^**^	0.936^**^	−0.746^**^	−0.917^**^	0.910^**^	0.447^*^	0.780^**^	0.157	1

* *Means p < 0.05*,

** *means p < 0.01*.

### Thermal Processing Hazards Content and Sensory Characteristics of Traditionally Baked Biscuits

The content of TPHs, including MG, 3-DG, AM, and HMF, and sensory characteristics, including AW, texture, and color, are shown in Table 3.

**Table 3:** The THPs content and AW, texture, and color characteristic in a traditionally baked biscuit.

**MG (mg/kg)**	**3-DG (mg/kg)**	**AM (mg/kg)**	**HMF (mg/kg)**	**AW**	L*	a*	b*	**Hardness (g)**	**Fracturability (mm)**
0.372 ± 0.013	4.524 ± 0.093	1.675 ± 0.130	0.904 ± 0.101	0.321 ±	74.62 ±	4.32 ±	30.45 ±	3,868.529 ±	5.333 ±
				0.017	0.08	0.04	0.08	135.198	0.260

### Sensory Evaluation

The fuzzy matrices for traditionally baked biscuits (190°C for 7 min; R_1_) and microwave baked biscuits (440 W for 3 min; R_2_) were as follows:


$${\text{R}}_{1}=\left|\begin{array}{cccc} 1 & 0 & 0 & 0\\ 0.3 & 0.4 & 0.2 & 0.1\\ 0.4 & 0.3 & 0.2 & 0\\ 0.5 & 0.3 & 0.2 & 0 \end{array}\right|;\;{\text{R}}_{2}=\left|\begin{array}{cccc} 0.9 & 0.1 & 0 & 0\\ 0.5 & 0.3 & 0.2 & 0\\ 0.6 & 0.2 & 0.2 & 0\\ 0.4 & 0.4 & 0.2 & 0 \end{array}\;\right|$$

Sensory attributes and preference degrees are shown by the vertical and horizontal components, respectively. Hence, the evaluation results were as follows:


$${\text{Y}}_{1}=\left(0.2,\;0.3,\;0.2,\;0.3\right)\times R_{1}=|\begin{array}{c} 0.52\&0.27\&0.16\&0.03 \end{array}\;|$$

$${\text{Y}}_{2}=\left(0.2,\;0.3,\;0.2,\;0.3\right)\times R_{2}=|\begin{array}{c} 0.57\&0.27\&0.16\&0 \end{array}\;|$$

Then, the defuzzification process was performed. According to the elements of excellent, good, fair, and poor with the score of 4, 3, 2, and 1, respectively, the total score of traditionally baked biscuits and microwave baked biscuits were: S_1_ = 3.24 and S_2_ = 3.41.

## Discussion

αDCs are reactive intermediates of the Maillard reaction that are induced by the thermal processing of foods; moreover, they are regarded as the precursors of flavor substance and color (22, 23). As αDCs are mutagenic and carcinogenic (24), their presence in foods is a risk to human health. Furthermore, αDCs are the precursors of various of TPHs (25); hence, the content of αDCs in foods should be seriously considered, especially the main αDCs formed in foods, such as MG and 3-DG. The generation of MG and 3-DG followed an increasing trend in microwave-baked biscuits at 440 W, but there was no significant difference in the increase of 3-DG. The content of MG and 3-DG significantly (*p* < 0.05) increased with the increase in microwave time at a power of 480 and 520 W. The results showed that the heat energy generated by microwave baking at 440 W did not reach the activation energy that could generate more MG and 3-DG. The Maillard reaction is a series of chain reactions; the formation of MG occurs through the retroaldolization of 3-DG (26). MG content is linearly related (*r* = 0.881, *p* < 0.01) to 3-DG content, showing that the MG formed in microwave-baked biscuits may be mainly derived from 3-DG.

The processing conditions that are conducive to the formation of DCs also facilitate the generation of AM and HMF (16). However, their formation in microwave-baked biscuits has been rarely reported. We found that the trend in the change of AM and HMF content was consistent with that of MG and 3-DG in microwave-baked biscuits. Furthermore, the correlation coefficients of MG with AM and HMF were 0.879 and 0.944 (*p* < 0.01), which was greater than the correlation coefficients of 3-DG with AM (*r* = 0.822, *p* < 0.01) and HMF (*r* = 0.910, *p* < 0.01). Some studies reported that αDCs could promote the formation of AM through the reaction of Strecker degradation (27, 28). In the Maillard reaction simulation system of glucose and asparagine, it was found that nearly 80% of MG was involved in the formation of AM as a precursor of AM (29). A study of honey and beer reported that 3-DG was transferred into HMF (30, 31). In a model Maillard system, HMF significantly promoted the formation of AM during heating (32). Several studies found that both MG and HMF participated in the formation of acrylamide in a Maillard simulation system or a food matrix (16, 32). From the above literature reports, it can be determined that 3-DG may directly or indirectly participate in the formation of the other three TPHs; therefore, 3-DG is significantly positively correlated with the other three TPHs. HMF, as a main product of hexose dehydration, has a lower melting point, which can more readily facilitate the formation of AM during heating (32). AM content is also linearly related (*r* = 0.809, *p* < 0.01) to HMF content. This showed that the increase in microwave power and the extension of microwave time promoted the formation of AM and HMF and their precursors.

AW is an index used to evaluate the free moisture that can participate in the Maillard reaction in the food systems. It is a key factor influencing the formation of TPHs (33, 34). The mobility or solubility of molecules is hindered at lower AW levels; conversely, the rate of the Maillard reaction decreased owing to the dilution of reactants with a higher AW level (35). However, the existing literature only considers the correlation between AW and AM and or HMF and ignores the correlation with precursor substances. We found that AW gradually decreased with the increase in microwave power and the extension of microwave time and that AW was negatively correlated (*p* < 0.01) with the four TPHs, with correlation coefficient in the following order: AM > MG > 3-DG > HMF. When AW is below 0.32, the levels of the four TPHs rise sharply. Ait-Ameur et al. (36) found that HMF was closely related to AW in traditional baked cookies systems. 3 mol of water was released when 1 mol HMF was formed. The AW range from 0.5 to 0.7 is known to facilitate the formation and accumulation of HMF. However, in the microwave-baked biscuit, this range did not elicit a similar phenomenon. This may be due to the different heating principles that had a different influence on the moisture migration. Similar results were found in fried potato chips, in which the formation of AM was highly related to AW (37). In cookies, an increase in HMF formation was found as AW decreased, and when the AW was below the critical point of 0.4, the HMF content increased greatly (38).

Color is the first parameter by which consumers judge food quality. L^*^, a^*^, and b^*^ color system parameters have been extensively used to analyze the significance of correlations between the surface color and the AM concentration in different foods (39–41). Gökmen et al. (19) developed a prediction model of AM content in traditional baked cookies based on computer visual image analysis algorithms and found that there was a significantly positive correlation (*r* = 0.946, *p* < 0.01) between the browning rate of biscuits and the amount of AM produced, the browning rate was calculated based on the data of L^*^, a^*^, and b^*^ values. The existing literature also ignored the correlation analysis between color and precursor substances. According to the Pearson analysis, there was a negative correlation coefficient between L^*^ and the MG, 3-DG, AM, and HMF, in the ranges from −0.803 to −0.985 (*p* < 0.01), and a positive correlation coefficient between a^*^ and MG, 3-DG, AM, HMF, and the total TPHs range from 0.790 to 0.986 (*p* < 0.01). The order of the correlation of L^*^ and a^*^ with the TPHs was the same: HMF > MG > 3-DG > AM. Compared with L^*^and a^*^, b^*^ is more poorly correlated with the formation of TPHs. L^*^ values are highly related to the degree of nonenzymatic browning; for example, a low L^*^ value means a greater degree of browning on the food surface (42). An increase in the a^*^ value indicates a redder shade and a greater degree of browning. Both the L^*^ and a^*^ values proved that the increase in microwave power and the extension of microwave time increased the degree of browning in microwave-baked biscuits.

Hardness and fracturability are two representative parameters for evaluating biscuit texture. The results showed that hardness was increased as the microwave time was prolonged and the microwave power was increased and was mainly positively correlated (*p* < 0.01) with the four TPHs. The texture of biscuits is mainly affected by the changes in the spatial structure of starch, protein, and moisture (43). Through the comprehensive analysis of the above sensory indicators, AW was shown to be mainly affected by microwave power and time; the texture was mainly affected by the spatial structure of starch, protein, and moisture; and color provided a comprehensive characterization of the influence of the temperature, time, AW, and reaction substrate concentration on the formation of THPs in the microwave-baked biscuit system (44). Therefore, color is the optimal sensory index, indicating the degree of browning and the formation of TPHs in microwave-baked biscuits, which was consistent with the order that the correlation between sensory characteristics and the formation of TPHs was: L^*^ > a^*^ > hardness > AW > b^*^ > fracturability.

Furthermore, given that texture cannot directly influence the formation of THPs, the biscuits baked in the microwave at 440 W for 3 min (with no significant difference in texture) were chosen for comparison with the traditionally baked biscuits. The contents of MG, 3-DG, AM, and HMF were decreased 60.75, 30.19, 30.87, and 61.28%, respectively. However, the sensory scores of traditionally baked biscuits were slightly higher than those of microwave-baked biscuits. This is mainly because the sensory evaluation integrates multiple evaluation attributes. The fuzzy matrix results showed that the difference in preference degree is mainly apparent in taste and color. Compared with microwave-baked biscuits, traditionally baked biscuits have a more obvious color, as characterized by lower L^*^ value, and larger a^*^ and b^*^ values. According to the correlation analysis, changes in these factors are more conducive to the formation of TPHs. This shows that, based on the same texture conditions, the analysis of the correlation between sensory characteristics and TPHs content in microwave-baked biscuits is also suitable for traditionally baked biscuits. Therefore, it is feasible to analyze the influence of different heating principles on the formation of TPHs based on the same texture characteristics.

In this study, we clarified that the microwave power and time facilitated the formation of TPHs and their precursors and explained the role of sensory characteristics in the microwave baking process. Among them, L^*^value can be used to predict the TPHs in microwave-baked biscuits. Moreover, for the same texture conditions, microwave baking can reduce the generation of TPHs.

## Data Availability Statement

The original contributions presented in the study are included in the article/supplementary material, further inquiries can be directed to the corresponding authors.

## Author Contributions

SW and YZ: conceptualization, validation, and writing—review & editing. LD: methodology, formal analysis, and writing—original draft. CQ: investigation and visualization. FW and ZY: resources and data curation. All authors contributed to the article and approved the submitted version.

## Funding

This study was funded by the National Key R&D Program of China (2019YFC1606202).

## Conflict of Interest

The authors declare that the research was conducted in the absence of any commercial or financial relationships that could be construed as a potential conflict of interest.

## Publisher's Note

All claims expressed in this article are solely those of the authors and do not necessarily represent those of their affiliated organizations, or those of the publisher, the editors and the reviewers. Any product that may be evaluated in this article, or claim that may be made by its manufacturer, is not guaranteed or endorsed by the publisher.
